# Coexistence of Ankylosing Spondylitis and Hereditary Multiple Exostoses:Coincidence or Association

**DOI:** 10.5812/iranjradiol.4242

**Published:** 2014-01-30

**Authors:** Abdolrahman Rostamian, Hamed Mazoochy, Shafieh Movassaghi, Seyed Mohammad Javad Mortazavi, Elham Sadeghzadeh, Fatemeh Shahbazi, Hossein Ghanaati

**Affiliations:** 1 Department of Rheumatology, Vali-Asr Hospital, Imam Khomeini Hospital Complex, Tehran University of Medical Sciences, Tehran, Iran; 2 Center for Research on Occupational Disease, Tehran University of Medical Sciences, Tehran, Iran; 3 Department of Orthopedics, Imam Khomeini Hospital Complex, Tehran University of Medical Sciences, Tehran, Iran; 4 Department of Biology, Payame Noor University, Karaj, Iran; 5 Advanced Diagnostic and Interventional Radiology Research Center (ADIR), Imam Khomeini Hospital Complex, Tehran University of Medical Sciences, Tehran, Iran

**Keywords:** Spondylitis, Ankylosing, Exostoses, Multiple Hereditary

## Abstract

Coexisting ankylosing spondylitis and hereditary multiple exostoses have rarely been reported (three patients) previously. A 27-year-old man with hereditary multiple exostoses is presented as a fourth report. At the age of 15 years, the patient had multiple exostoses around the knee, ankle and shoulder joints. He was diagnosed with ankylosing spondylitis 3 years ago. The patient’s sister and his 3 brothers also have multiple exostoses without any family history of spondyloarthropathy or inflammatory arthritis. The aim of this report is to discuss an interesting coexistence of these two diseases. The increasing number of reported patients who have a coexistence of these two diseases might suggest that the association of these two diseases is stronger than a coincidence.

## 1. Introduction

Ankylosing spondylitis (AS) is a chronic inflammatory rheumatic disease of the spine and sacroiliac joints that is present mainly in young people. AS has a prevalence of 0.5-1.9% for all types of spondyloarthritis. Clinical features of this disease are axial skeletal ankylosis, inflammation at the insertions of tendons, pain and stiffness of the back, and radiological arthritis changes in the sacroiliac joints and frequently in the spine. The significant role of genetic factors in the development of AS has been identified (1-4). AS shows a striking correlation with antigen HLA-B27 (5). Hereditary multiple exostoses (HME) is a genetically heterogeneous disorder that is correlated with mutations in the exostoses gene family (EXT1, EXT2) (6). The incidence of HME is 1 per 50,000. Exostoses appear from early childhood and usually continue to grow until the end of puberty, affecting longer bones more, as it appears more frequently in the distal end of the femur (90%), proximal end of the tibia (84%), proximal end of the fibula (76%), proximal end of the humerus (72%) and to a lesser extent in the vertebrae (7%) and the sternum (1%) (7). The frequency of HLA-antigens in HME patients is not clear.

To our knowledge, only three cases of AS associated with exostoses have been previously reported (8-10). The first case,a Taiwanese young man with amphetamine addiction had a strange combination of exostoses, AS, alpha thalassemia and thrombocytopenia (9). The second and third cases, a 25 and a 50-year-old (Turkish men), showed a coexistence of AS and HME (8, 10). It persuades us to report another case of HME coexisting with AS. In this paper, we present a patient with HME who was diagnosed with AS based on clinical and laboratory findings.

## 2. Case Presentation

**Figure 1.**
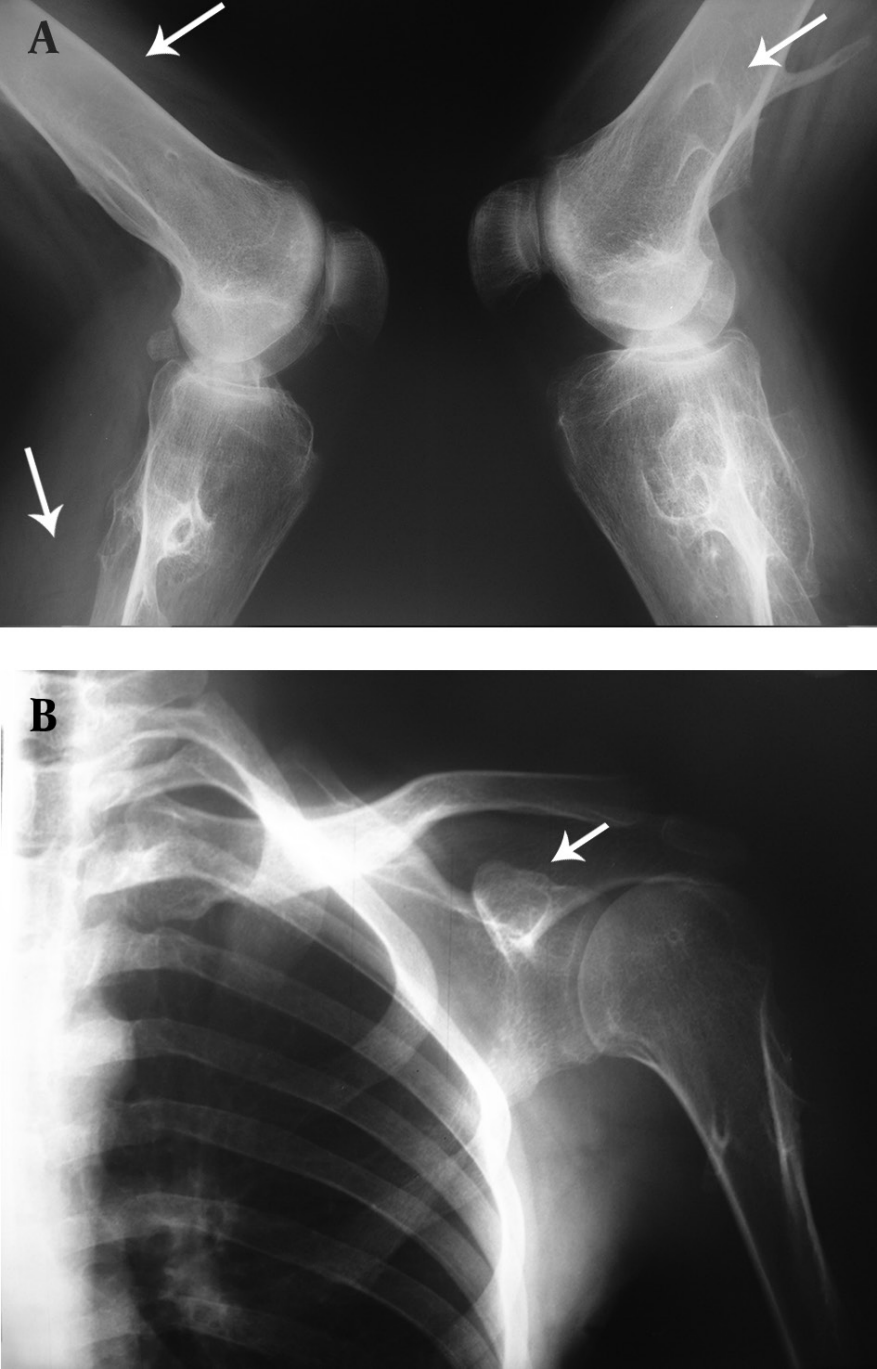
A, Lateral X ray of the knee shows radial multiple exostoses around the knee (thin white arrow). B, Anteroposterior X ray of the shoulder shows exostoses around the shoulder joint (thin white arrow).

We report a 27-year-old man who was referred to Imam Khomeini Hospital with chief complaints of pain and an intensive restriction in the range of motion (ROM) in the bilateral hip joints. He was diagnosed with HME 12 years ago while he had hip trauma, and multiple exostoses around the knee, ankle and shoulder joints were found in the obtained X rays (Figure 1 A and B). He was diagnosed as AS 3 years ago due to low back pain (LBP) accompanied with significant morning stiffness that lasted about 1.5 h together with laboratory and radiological findings. He was diagnosed as AS based on New York criteria including clinical criteria; a) low back pain more than 3 months improved by exercise and not relieved by rest, b) limitation of lumbar spine motion in sagittal and frontal planes and limitation of chest expansion, and radiologic criteria of bilateral sacroiliitis grade 2 or more (11).

The patient’s sister and his 3 brothers have multiple exostoses without any family history of spondiloarthropathy or inflammatory arthritis. He has been taking sulfasalazine 1500 mg per day and indomethacin 75 mg twice a day because the bilateral hip joint ROM limitation had caused motion dysfunction. Flexion arc was limited to 30 degrees with a maximum flexion of 60 degrees. Motion was also restricted in the adduction-abduction arc as well as the rotator arc. Specific examination for AS including the Schober test and chest expansion was positive. Lateral bending was limited.The laboratory tests revealed mild anemia (Hb: 11 mg/dl, Hct: 37%, MCV: 79 fL) and a high ESR rate (93 mm/h). C-reactive protein and HLA-B27 were positive, but rheumatoid factor, ANA, viral markers and the Wright test were negative.

**Figure 2.**
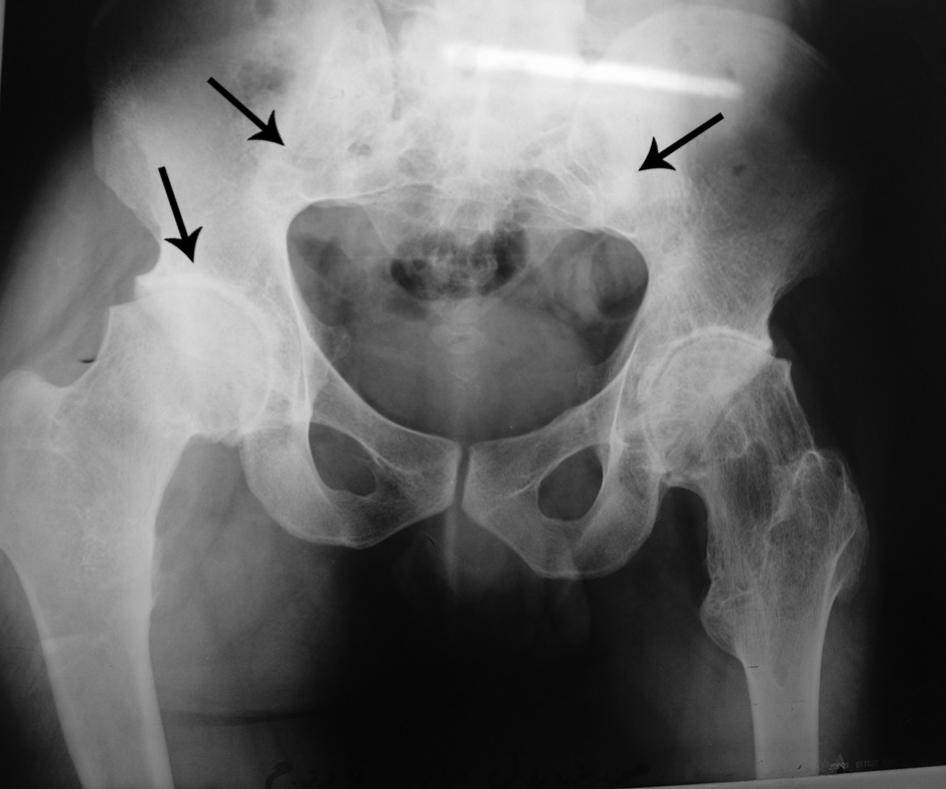
Reduction of the hip joint spaces and bilateral sacroiliitis. AP radiograph of the hip shows symmetrical narrowing of both hip joint spaces (black arrow), bilateral sacroiliac joint erosions and iliac side subchondral sclerosis (bilateral sacroiliitis, the arrows at the top).

**Figure 3.**
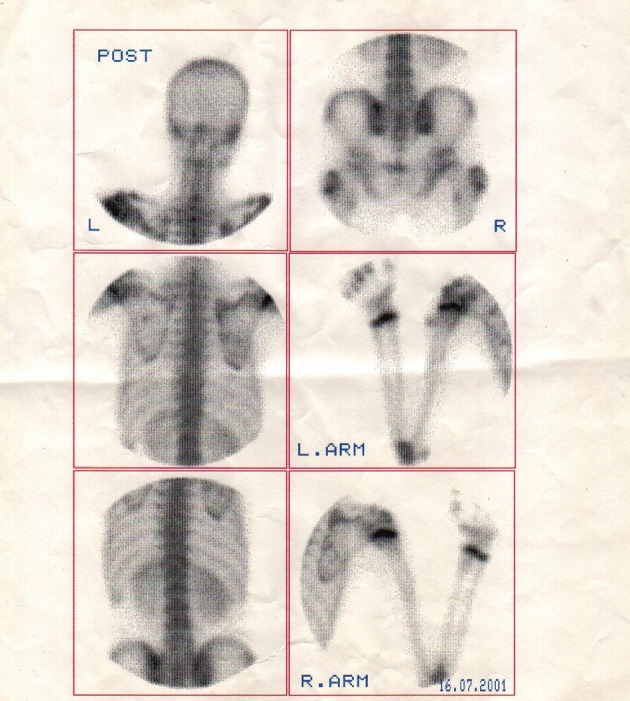
Whole body bone scan revealsincreased uptake in the metaphysis of different long bones.

**Figure 4.**
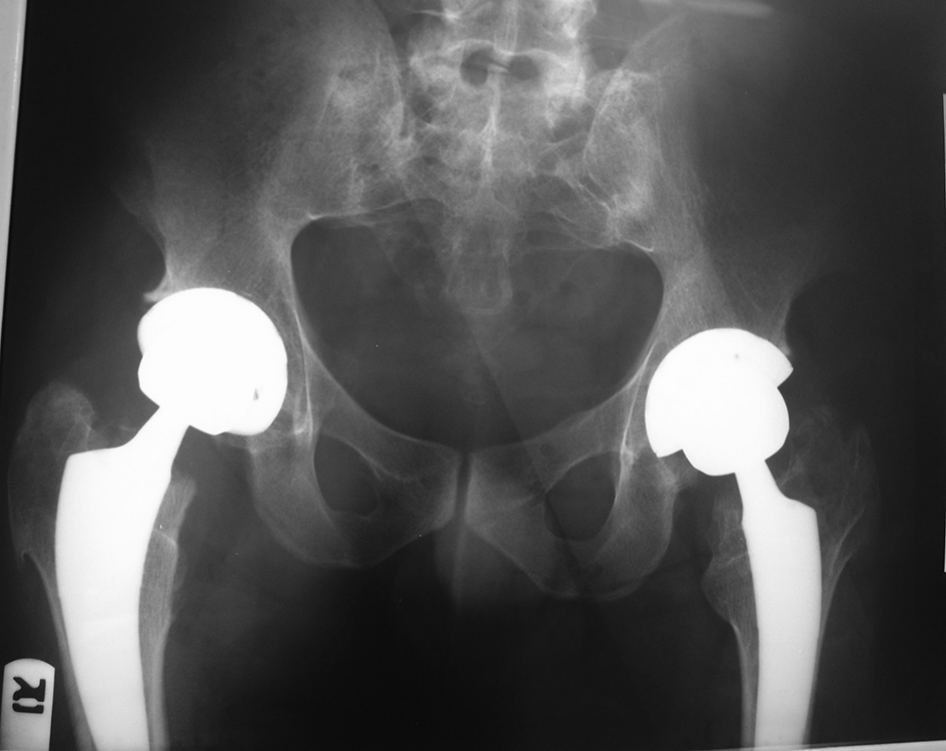
Total hip replacement (arthroplasty)

Multiple exostoses were seen around the knee, ankle and shoulder joints in the X rays (Figure 1 A and B). Both sacroiliac joints were fused completely and the joint spaces of both hips were narrowed (Figure 2). A whole body bone scan showed increased activity in the metaphysis of multiple long bones that supported multiple exostoses (Figure 3). Hip MRI revealed decreased joint distance and obliteration of both sacroiliac joints with subchondral bone changes due to sacroiliitis. Dueto severe bilateral restricted hip motion, the patient had impaired functional activity. Bilateral total arthroplasty was performed (Figure 4).

## 3. Discussion

The first, second and third reports of coexistence of HME and AS were published in 1996, 1999 and 2006, respectively and this case report is the fourth case. With only four cases of coexistence of HME and AS so far, it seems that either association of HME and AS is a rare entity or it is a coincidence. The coexistence of two diseases is unlikely to be fortuitous. The increase in the number of reported patients who have a coexistence of these two diseases might suggest that the association of these two diseases is stronger than a coincidence. However, the role of genetic history, gene expression and a pre-existing inflammatory process or tissue destruction should be under consideration. To obtain clear and conclusive information and to reach a better understanding of this association, further study of the genetic history is suggested.

Coexistence of two diseases is encountered in rheumatology practice, particularly the association of AS with different diseases such as acromegaly, Behçet's disease and gouty arthritis in three Turkish patients (12-14) and coexisting AS and HME in 4 men [two Turkish men, one Taiwanese (8-10) and one Persian]. From this point of view, it may be suggested that the coexistence of AS with other diseases has occurred more in Asia and particularly in Turkey. It may be interesting to study all AS patients in Turkey.

Similar to the Taiwanese patient (with thrombocytopenia), the Persian patient has shown anemia, both idiopathic thrombocytopenia and anemia are autoimmune disorders. It is possible that the autoimmune disorders present with AS and their accompaniments trigger the expression of the exostoses gene family in Asian descent. Therefore, further comprehensive studies should be scheduled to assess the coincidence or association of these diseases.
